# Impact of heatwaves on all-cause mortality in India: A comprehensive multi-city study

**DOI:** 10.1016/j.envint.2024.108461

**Published:** 2024-01-26

**Authors:** Jeroen de Bont, Amruta Nori-Sarma, Massimo Stafoggia, Tirthankar Banerjee, Vijendra Ingole, Suganthi Jaganathan, Siddhartha Mandal, Ajit Rajiva, Bhargav Krishna, Itai Kloog, Kevin Lane, Rajesh K Mall, Abhiyant Tiwari, Yaguang Wei, Gregory A. Wellenius, Dorairaj Prabhakaran, Joel Schwartz, Poornima Prabhakaran, Petter Ljungman

**Affiliations:** a Institute of Environmental Medicine, Karolinska Institutet, Stockholm, Sweden; b Center for Climate and Health, Boston University School of Public Health, Boston, MA, United States; c Department of Epidemiology, Lazio Region Health Service /ASL Roma 1, Rome, Italy; d Institute of Environment and Sustainable Development, Banaras Hindu University, Varanasi, India; e Office for National Statistics, Wales, Newport, United Kingdom; f Centre for Chronic Disease Control, New Delhi, India; g Ashoka University, Sonipat, India; h Centre for Policy Research, New Delhi, India; i Ben-Gurion University of the Negev, Beer-Sheva, Israel; j Department of Environmental Medicine and Public Health, Icahn School of Medicine at Mount Sinai, New York, NY, USA; k DST-Mahamana Center of Excellence in Climate Change Research, Institute of Environment and Sustainable Futures Collaborative, New Delhi, India; l NRDC India, New Delhi, India; m Department of Environmental Health, Harvard T.H. Chan School of Public Health, Boston, MA, USA; n Department of Cardiology, Danderyd Hospital, Stockholm, Sweden

**Keywords:** India, Heatwaves, Mortality, Attributable deaths, Climate change

## Abstract

**Background:**

Heatwaves are expected to increase with climate change, posing a significant threat to population health. In India, with the world’s largest population, heatwaves occur annually but have not been comprehensively studied. Accordingly, we evaluated the association between heatwaves and all-cause mortality and quantifying the attributable mortality fraction in India.

**Methods:**

We obtained all-cause mortality counts for ten cities in India (2008–2019) and estimated daily mean temperatures from satellite data. Our main extreme heatwave was defined as two-consecutive days with an intensity above the 97th annual percentile. We estimated city-specific heatwave associations through generalised additive Poisson regression models, and *meta*-analysed the associations. We reported effects as the percentage change in daily mortality, with 95% confidence intervals (CI), comparing heatwave vs non-heatwave days. We further evaluated heatwaves using different percentiles (95th, 97th, 99th) for one, two, three and five-consecutive days. We also evaluated the influence of heatwave duration, intensity and timing in the summer season on heatwave mortality, and estimated the number of heatwave-related deaths.

**Findings:**

Among ~ 3.6 million deaths, we observed that temperatures above 97th percentile for 2-consecutive days was associated with a 14.7 % (95 %CI, 10.3; 19.3) increase in daily mortality. Alternative heatwave definitions with higher percentiles and longer duration resulted in stronger relative risks. Furthermore, we observed stronger associations between heatwaves and mortality with higher heatwave intensity. We estimated that around 1116 deaths annually (95 %CI, 861; 1361) were attributed to heatwaves. Shorter and less intense definitions of heatwaves resulted in a higher estimated burden of heatwave-related deaths.

**Conclusions:**

We found strong evidence of heatwave impacts on daily mortality. Longer and more intense heatwaves were linked to an increased mortality risk, however, resulted in a lower burden of heatwave-related deaths. Both definitions and the burden associated with each heatwave definition should be incorporated into planning and decision-making processes for policymakers.

## 1. Introduction

One of the most direct impacts of climate change globally is increased variability in temperature distribution, with extreme temperature events occurring more frequently at either end of the temperature distribution (IPCC, 2023). The frequency of heatwaves has increased in recent years, consistent with anthropogenic climate change (IPCC, 2023). Evidence suggests that temperature extremes are a leading cause of weather-related mortality worldwide (Abbafati et al., 2020). The burden of disease associated with extreme heat is particularly pronounced in Low and Middle-Income Countries (Zhao et al., 2021) such as India, where extreme environmental exposures intersect with unplanned urbanization, poor-quality housing, declining urban green cover and other vulnerabilities in the world’s most populous country (Mazdiyasni et al., 2017). A national assessment of climate change conducted by the Indian government predicts increasing temperatures in India throughout the 21st century with an increase in extreme heat events (Im et al., 2017; INCCA, 2010).

Exposure to extreme heat may cause illness and mortality through a variety of biological mechanisms; importantly, there are physiological mechanisms triggered by heat exposure (i.e., ischemia, heat cytotoxicity, inflammatory response, disseminated intravascular coagulation, and rhabdomyolysis) as well as vital organs that can be critically impacted (i.e., brain, heart, intestines, kidneys, liver, lungs, and pancreas) (Mora et al., 2017). Heat-related morbidity and mortality can be caused by direct effects of exposure to extreme heat, including for example a spectrum of heat related illnesses from heat exhaustion to heat stroke (Mora et al., 2017). Equally challenging from a public health perspective are the indirect effects of extreme heat exposure, occurring when heat exposure stresses underlying physiological systems and results in other specific manifestations such as renal insufficiency, acute cerebrovascular and cardiovascular disease, and exacerbations of pulmonary disease. All-cause mortality represents a health endpoint that can illustrate both the direct and indirect impacts of extreme heat exposure.

A heatwave is a prolonged period of unusually and excessively hot weather, which may also be accompanied by high humidity. Definitions vary regionally, in both public health and policy literature, in part because a heatwave is measured relative to the usual weather in the area and relative to normal temperatures for the season, and in part because there is no single best indicator from a public health perspective (Xu et al., 2016). According to the Indian Meteorology Department (IMD) (IMD, 2023), any day in India may be a candidate for a heatwave declaration when the maximum temperature of a station reaches at least 40 °C or more for plains and at least 30 °C or more for hilly regions. Heatwave decisions are made according to 2 criteria – based on a departure from the normal temperature, or an absolute temperature threshold. For departure from the normal, a heatwave is declared when the maximum temperature is more than 4.5 °C over the 30-year average historical maximum temperature (IMD, 2023). If the actual maximum temperature is above 45 °C, a heatwave is declared irrespective of the normal historical maximum temperature (IMD, 2023).

India has had several heatwaves which have increased in frequency during the last decades (Singh et al., 2021). Most notably, in May 1998, India experienced a severe heatwave over a 2-week period considered to be the worst among the preceding 50 years (Kalsi and Pareek, 1998). The following year, a similar record-breaking event occurred in north-west and central India. During the summer of 1999, India experienced unprecedented heat in April, with maximum temperatures of 40 °C or above for more than 14 days (Kalsi and Pareek, 2001). Another heatwave in 2003 was estimated to have caused more than 3,000 deaths in Andhra Pradesh (Solomon, 2007). In May 2010, a heatwave in Ahmedabad (Gujarat) killing approximately 1300 deaths (Azhar et al., 2014), triggered the implementation of several heat action plans intended to mitigate the impacts of extreme heat nationwide (Knowlton et al., 2014). In recent years, such as in 2016 (CNN, 2016), 2018 (WEF, 2018), 2019 (BBC, 2019) and 2023 (The Guardian, 2023) extreme heatwaves have been observed across India, but few studies have evaluated the health impact of these extreme events across multiple Indian cities, and those studies focus on earlier years of data (Azhar et al., 2014; Nori-Sarma et al., 2019b, 2019a; Rathi et al., 2023). Differences in demographics and socioeconomic characteristics among different communities may influence the vulnerability and adaptive capacities to heatwave events, highlighting the importance of community-specific analyses and interventions (Dimitrova et al., 2021).

While the health impacts of heatwaves, and particularly the mortality impacts, have been explored on a global scale in many regions of the world (Dimitrova et al., 2021), there is limited information on specific impacts and characteristics of the relationship between multiple days of consecutive excess heat exposure and health impacts in India (Azhar et al., 2014; Nori-Sarma et al., 2019b, 2019a). Heat-health promotion strategies in India, including extreme heat early warning and preparedness systems have only recently been made a matter of policy at the city government level (Pillai and Dalal, 2023; Knowlton et al., 2014). We sought to investigate the association between elevated temperatures over single and multiple day periods (hereafter referred to as a “heatwave”) and mortality risks and quantified the mortality fraction attributable to heatwaves across ten cities in India.

## 2. Methods

### 2.1. Study population

**Fig. 1.**
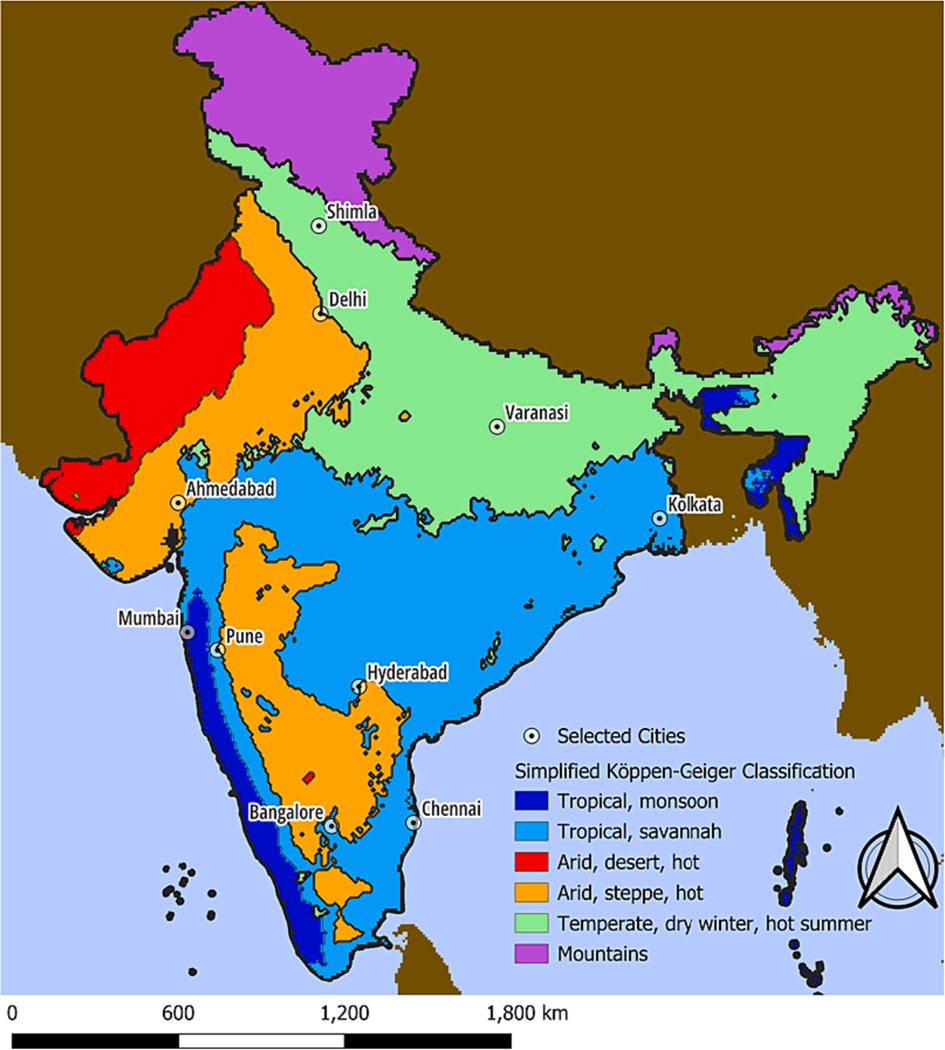
Study cities in India according to a simplified Köppen-Geiger climate zone classification. Footnote: this is a derivative map from the Köppen Geiger classification(Beck et al., 2018). We removed some of the categories which were in small quantity and were not possible to significantly differentiate enough (e.g. Temperate, dry winter, warm summer was subsumed into Temperate, dry winter, hot summer).

This study included daily all-cause mortality counts from the birth and death registers of 10 municipal corporations in India including Ahmedabad, Bangalore, Chennai, Delhi, Hyderabad, Kolkata, Mumbai, Pune, Shimla, and Varanasi (Table 1). The 10 cities are in different Koppen-Geiger climate zone classifications of India: arid, steppe, hot ¨ (Ahmedabad), tropical monsoon (Mumbai), tropical savannah (Bangalore, Chennai), and temperate, dry winter, hot summer (Shimla, Varanasi), and composite of climate zones (Delhi, Hyderabad, Pune) (Fig. 1). Data from the largest cities such as Delhi, Chennai, Kolkata and Mumbai covered larger periods from 2008 till 2015–2019 while datasets from other smaller cities ranged mainly between 2008 and 2012 (Table 1).

### 2.2. Exposure assessment: Temperature

Daily mean temperatures were obtained from the European Centre for Medium-Range Weather Forecasts (ECMWF) at a resolution of 28 km × 28 km resolution (0.25° _×_ 0.25°). Data was freely downloaded from the Copernicus Climate Data store (https://cds.climate.copernicus.eu/). In this study, we estimated daily mean temperatures of the grid cells of ECWMF within each of the 10 municipal corporations’ boundaries. Since we only had maximum temperature from meteorological stations for 58 % of the data, we used daily mean temperature which had 100 % coverage for the days of outcome data within our dataset. The correlation between daily maximum temperature and daily mean temperature was 0.94 for observations where we had data for both. For this reason, we only focused on mean temperature facilitating a longer record of data. In addition, we included daily dew point temperatures obtained from ECMWF as a proxy of relative humidity. As mean temperature from ECMWF is used to estimate dew point temperature and to limit collinearity, we regressed daily mean temperature on the dew point temperatures, and used the residuals of that model in our regression analysis (Brooke Anderson and Bell, 2011).

### 2.3. Heatwave definition

A heatwave is defined as a prolonged period of unusually and excessively high temperatures for a given area. In our primary analyses, our main extreme heatwave definition was defined as two consecutive days with an intensity above the 97th annual percentile. This definition served the purpose of capturing hot and sustained weather episodes that are rare but not extreme outliers. We further identified heatwaves as one, two, three and five consecutive days with an intensity above the 95th, 97th and 99th annual temperature percentile based on the scientific literature (Xu et al., 2016). We included one consecutive day above a specific percentile as the India Meteorological Department (IMD) allows one single day to declare a heatwave. In our analyses we only focused on mean temperature as maximum temperature was not available for all data points; while this prevents an analysis of a heatwave definition that matches the IMD heatwave definition, it is consistent with many international studies (Xu et al., 2016).

### 2.4. Statistical analyses

#### 2.4.1. Estimating the health effects of heatwaves

We applied a two-stage analysis approach to evaluate the effects of heatwave days on daily mortality counts using a time-series design. In the first stage, we fit quasi-Poisson generalized additive regression models to estimate city-specific associations. The models were adjusted for a penalized spline smooth function of calendar day with nine degrees of freedom (*df*) per year to account for seasonality and underlying time-trends, an indicator of day-of-week to account for weekly variations, a natural spline function with 4 *df* for dew point temperature residuals (lag 0–1) and particulate matter less than 2.5 μm in diameter (PM_2.5_) (lag 0–1). City-wide daily PM_2.5_ levels were obtained from a novel hybrid nationwide spatiotemporal model (Mandal et al., 2022). We used the same day of heatwave definition to estimate the effect on daily mortality, based on prior studies illustrating the acute impacts of heat on health. The effect estimates are reported as the percentage change in daily mortality, with 95 % confidence intervals (CI), for comparing heatwaves days versus non-heatwave days. In the second stage, we applied a random-effect *meta*-analytical model to pool the city-specific estimates of associations of heatwaves with mortality. We computed I^2^ statistics and Cochran’s Q test to evaluate the between-city heterogeneity.


(1) $$\mathrm{Increase}=\left(\frac{\mathrm{average of daily mean temperature of HW}-97^{\mathrm{th}}\mathrm{percentile annual temperature}}{\mathrm{average of daily mean temperature of HW}}\right)\times 100$$

#### 2.4.2. Heatwave characteristics

We further evaluated the modification of heatwave-mortality estimates by heatwave characteristics based on our main heatwave definition. For this, we included each individual heatwave as a unique identifier in the model and estimated the mortality effect of each individual heatwave for each city and adjusted for the same confounders as specified previously. For each heatwave, we then calculated three different characteristics:

- Duration: the length in days for each specific heatwave.
- Intensity: estimated as the percentage increase of the average daily mean temperature of the specific heatwave compared to the 97th percentile of the specific city (equation (1)). Thus, 0 % intensity equals a mean temperature of exactly the 97th percentile. We did not apply absolute temperature (°C) increases because we had very different baseline temperature across the cities (Nori-Sarma et al., 2019a).
- Timing of the summer season: this was characterized as the days when the heatwave started within the summer season. The start of the summer season was considered 1st of March for all cities except Delhi and Varanasi which was 1st of April.

Once we obtained the individual associations between each specific heatwave definition and mortality, we applied a *meta*-analysis to evaluate the relationship between the characteristics of heatwaves and mortality (Brooke Anderson and Bell, 2011; Nori-Sarma et al., 2019a). We evaluated each characteristic individually in single exposure models and mutually adjusted all characteristics in one multiple exposure model in order to evaluate the independent effect of each characteristic. We included a random effect for each city and applied inverse-variance weighting to account for city-specific differences and the precision of the effect estimates, respectively. The obtained estimates would explain how the characteristics of each heatwave modify the associations between heatwaves and mortality.

#### 2.4.3. Attributable fraction

We further assessed the number of attributable deaths by heatwave definition and city. We used the relative risk estimates to determine the attributable risk percent, representing the proportion of mortalities attributed to heatwaves among all deaths (equation (2)). Next, this percentage was multiplied by the daily average expected deaths for the city and the number of heatwave days to obtain the total number of attributable deaths per heatwave definition. The methodology employed in this study is consistent with previous research that aimed to quantify the cumulative mortalities associated with heatwaves (Nori-Sarma et al., 2019b):

(2) $$D_{a}=\frac{N_{HW}^{c}}{year}\times D_{e}^{c}\times \left(\frac{RR_{HW}^{c}-1}{RR_{HW}^{c}}\right)$$

where $N_{HW}^{c}/\text{year}$ = Number of heatwave days per year, varies by heatwave definition (*HW)* and city c; $D_{e}^{c}$ = average number of expected daily deaths for city c ((annual mortality rate * city population)/365.24 days); $RR_{HW}^{c}$= city-specific heatwave relative risk. This equation includes both the amplitude of the effect of the heatwave and its prevalence.

### 2.5. Sensitivity analyses

We performed several sensitivity analyses to test the robustness of our results. We evaluated the effect of maximum temperature on daily mortality rather than using mean temperature where such data was available. To evaluate different adjustments for time trends, we applied different *df* (between 6 and 10 *df*/year) and a year indicator and harmonics (sine–cosine pair) which seemed to be more accurate to apply in lower spatial resolution exposure setting (Leung et al., 2023). Additionally, we removed the adjustments by dew points and PM_2.5_. Finally, we adjusted for a spline of daily mean temperature to separate the effect of heatwaves from the effects of single day temperatures.

## 3. Results

### 3.1. Heatwave descriptive definition

In this multi-city time-series analysis, we observed an average of 136 daily deaths across cities, ranging from 5 to 284 daily deaths in Shimla and Delhi, respectively (Table 1). When using our main heatwave definition (2-consecutive days surpassing the 97th annual temperature percentile), we identified a total of 168 heatwaves during the period ranging between 2008 and 2019. On average, there were 3 heatwaves per year across all cities. Hyderabad and Pune had the highest average number of heatwaves per year at 4.0 and 4.3, respectively, while Chennai and Varanasi had the lowest average at 2.2 each. Furthermore, Delhi and Shimla exhibited the highest “intensity” or percentage increase in temperature above the average of the daily mean temperature, with intensities of 3.0 % and 3.8 % respectively.

### 3.2. Associations between heatwaves and mortality

**Fig. 2.**
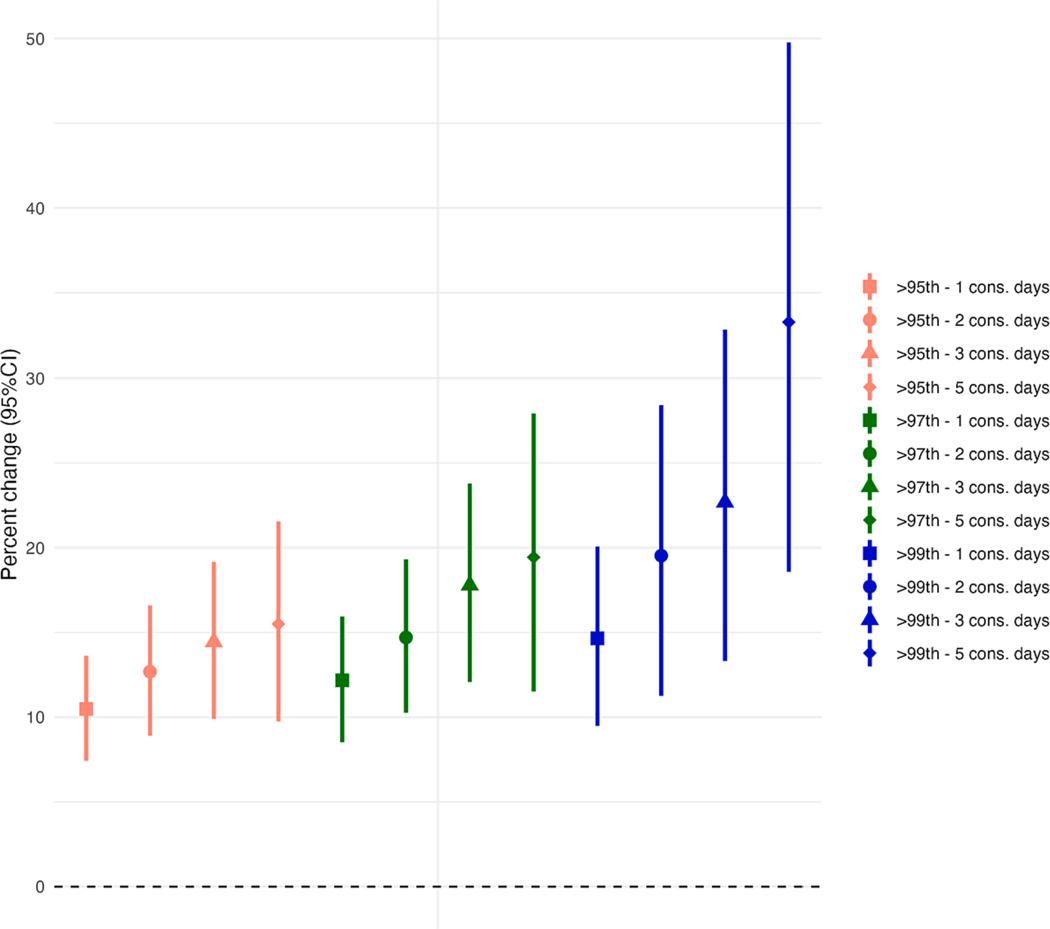
Pooled estimates of the percent increase (95% CI) in mortality risk due to multiple heatwaves definitions (numeric effect estimates are shown in Table S1) Abbreviation: HW, heatwave. Note: Estimates are provided as percentage change in mortality and 95% confidence interval comparing heatwaves vs non-heatwave days. Models were adjusted for a penalized spline smooth function of calendar day with nine degrees of freedom (*df*), an indicator of day-of-week, a natural spline function with 4 *df* for adjusted dew point temperature (lag 0–1, and air pollution (lag 0–1). Heatwaves above the 99th percentile for 5 consecutive days was estimated including 6 cities (Ahmedabad, Chennai, Delhi, Hyderabad, Kolkata, Varanasi).

Our main heatwave definition showed that a heatwave with daily mean temperature above the 97th percentile for 2 consecutive days was associated with a 14.7 % (95 %CI, 10.3; 19.3) increase in daily mortality (Fig. 2 and Table S1), comparing heatwave to non-heatwave days. The city-specific estimates showed large variations, ranging from 3.4 % (95 %CI, 0.8; 6.1) for Mumbai to 24.9 % (95 %CI, 21.7; 28.2) for Ahmedabad. We observed a higher effect size estimate for daily mortality using heatwave definitions with successively higher percentiles as cutoffs and longer duration (Fig. 2 and Table S1). As a comparison to our main definition above, we observed a lower (12.7 %; 95 %CI, 8.9; 16.6) and higher (19.5 %; 95 %CI, 11.3; 28.4) increase in daily mortality for 2 consecutive days above the 95th, and 99th percentile, respectively. Regarding heatwave duration, we observed that mean temperatures above 97th for a consecutive 1, 2, 3 and 5 days were associated with 12.2 % (95 %CI, 8.5; 15.9), 14.7 % (95 %CI, 10.3; 19.3), 17.8 % (95 %CI, 12.1; 23.8), 19.4 % (11.5; 27.9) increase in daily mortality, respectively. The strongest increase in daily mortality was 33.2 % (95 %CI, 18.6; 49.8) when mean temperatures were above the 99th percentile for at least 5 consecutive days, which only occurred in 6 cities (Ahmedabad, Chennai, Delhi, Hyderabad, Kolkata, Varanasi). Consistently across definitions, the strongest associations were observed in Ahmedabad and Varanasi, and the weakest in Bangalore and Mumbai.

### 3.3. Effect modification by heatwave characteristics

Restricting the analyses to the heatwaves of the main approach (97th percentile for at least 2-consecutive days), we found differential associations between heatwaves and mortality by the length, intensity, and day (timing) in the summer season (Table 1 and Figure S1). The longest heatwaves occurred in Ahmedabad (15 days), Chennai (21) and Varanasi (19), while the most intense heatwaves were observed in Delhi (7.5 % increase above the 97th percentile) and Shimla (7.1 %). Pune and Bangalore experienced the earliest heatwaves in the season, occurring around the end of March (Table 1 and Figure S1). When we assessed the associations between heatwave characteristics and mortality, we observed that a one percent increase in mean intensity of the heatwave was associated with an extra 3.8 % increase (95 %CI, 2.8; 4.7) in the estimated association between heatwaves and mortality (Table 2 and Table S2). The strongest association was found for Pune with an extra 6.8 % (95 %CI, 2.0; 11.7) increase in the effect on the heatwaves-mortality association. Combining all heatwaves across cities, we observed an association for an increase in the duration of the heatwave (1.4 % per 1 day increase in heatwave (95 %CI, 0.7; 2.0) but this was attenuated in the mutually adjusted model. The timing of the heatwave during the summer season did not affect the estimated risk of heatwaves on mortality.

### 3.4. Attributable deaths

**Fig. 3.**
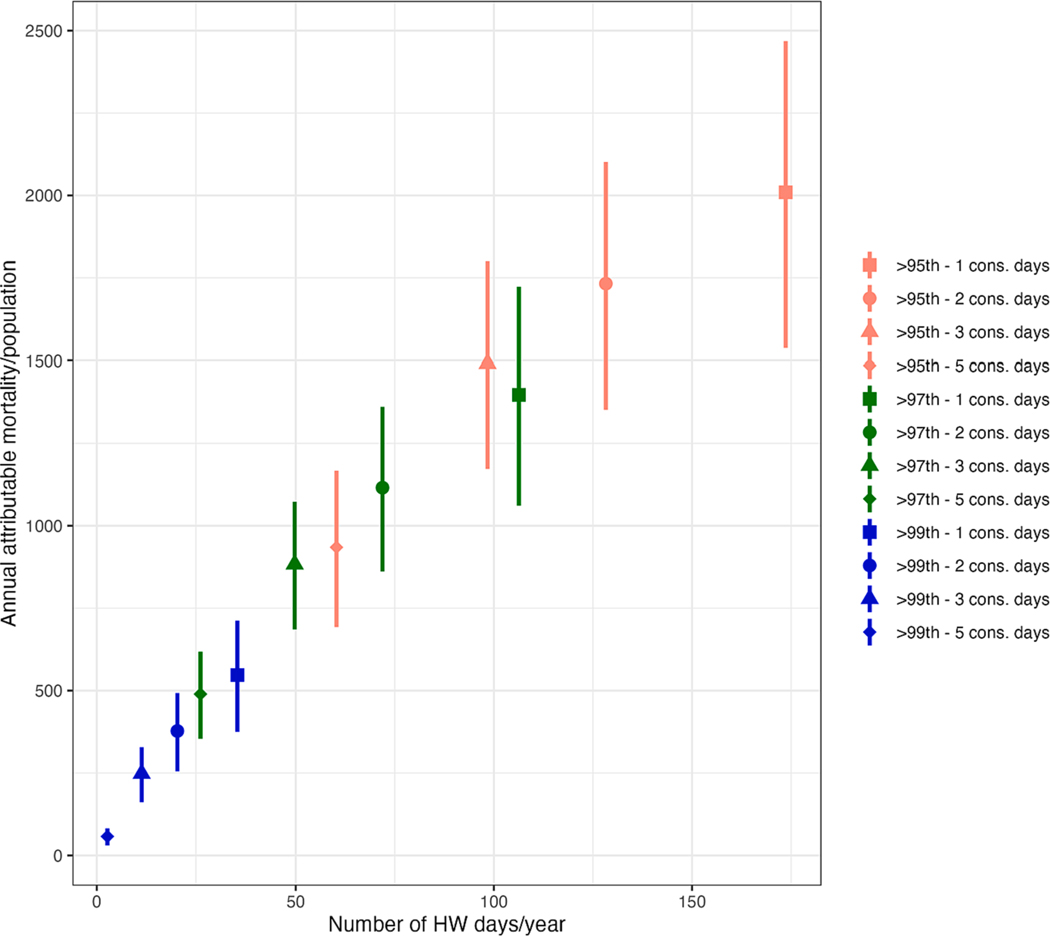
Comparison of attributable deaths across different heatwave definitions.

We observed an increase in the number of attributable deaths related to heatwaves when using shorter durations and heatwave definitions using lower percentile thresholds of daily temperature (Fig. 3). We estimated that around 1116 deaths annually (95 %CI, 861; 1361) across the 10 cities were attributable to heatwaves defined as 2-consecutive days above the 97th percentile. In contrast, 58 deaths (95 %CI, 30; 82) were attributed to heatwaves defined as at least 3 consecutive days above the 99th percentile. This trend was consistently observed across all cities. Higher annual attributable mortality was observed in Delhi, Ahmedabad and Chennai, whereas Shimla and Pune exhibited the lowest attributable mortality (Table S3).

### 3.5. Sensitivity analyses

In the sensitivity analyses, we observed slightly weaker associations when using maximum temperature in the heatwave definition rather than mean temperature, but the confidence intervals were largely overlapping (Supplementary Fig. S2). In addition, we observed almost identical effect estimates adjusting for different degrees of freedom per year for time trend and removing dew point and PM_2.5_ from the fully adjusted model (Supplementary Fig. S3). Adjusting for day of the year using a sine–cosine pair, the effect estimates slightly increased, but the confidence intervals also widened, overlapping with those from the main model. Furthermore, we aimed to separate the impact of heatwaves from the effects of single-day temperatures, to assess the added impacts of extended periods of extreme heat above the impacts of temperature. We found that the effect of heatwaves became less pronounced when considering shorter length of heatwaves (1 or 2 consecutive days above different percentiles) (Figure S3). However, as the length of heatwaves increased, we observed similar effects on mortality to those obtained in our main model.

## 4. Discussion

This study represents the largest multi-city time-series analysis conducted in India evaluating the associations between heatwaves and mortality, which is of particular significance due to the prevalence of extreme heat events in the country. We observed that heatwave episodes were associated with increased mortality risks across diverse cities, including major cities like Delhi, Mumbai, Kolkata and Chennai. These associations held true for all definitions of heatwaves. We further observed that longer and more intense heatwaves were linked to an increased risk of mortality. However, shorter and less intense heatwaves resulted in a higher burden of heatwave-related deaths, since more days qualified under the less stringent definition. Notably, even a single day of extreme heatwave conditions was found to elevate the risk of mortality. We found that on average, there were 1116 incremental deaths per year attributable to heatwaves in these 10 cities, which suggests a substantial burden of heatwaves, particularly if similar results were observed in the rest of India.

While previous research in India has predominantly explored the impact of increased temperatures on mortality, limited attention has been given to specifically investigating heatwaves and their consequences (Dimitrova et al., 2021). These studies have focused mainly on one specific city or region in India such as the extreme heatwave that occurred in Ahmedabad in 2010, where temperatures reached 46.8 °C, resulting in more than 1300 deaths of which 800 deaths occurred in a single week (Azhar et al., 2014). From our study, Ahmedabad emerged as one of the cities exhibiting the most pronounced health effects of heatwaves contributing to approximately 300 annual attributable deaths. We also observed similar results of heatwaves effects in a similar study in Varanasi (Singh et al., 2019). Another study explored the association between heatwaves and mortality in the northwest region of India (Nori-Sarma et al., 2019a). Their findings align closely with our own, as they reported an 18 % increased risk of mortality during heatwaves lasting two consecutive days above the 97th percentile. Our research yielded a comparable result, indicating a 15 % increase in mortality using the same definition, but our study included a variety of 10 cities across India from different climate zones. Further investigations are warranted to establish comparative analyses, focusing on cause-specific health outcomes, and targeting specific vulnerable populations by age, gender, and socioeconomic status.

The observed associations with heatwave mortality were impacted by the operational heatwave definition. We found an increased association in daily mortality as the heatwave definitions increased in intensity and in length based on specific annual temperature percentiles of daily mean temperature. These findings align with previous studies conducted worldwide that employed similar percentile definitions (Xu et al., 2016). Currently, there is no universally accepted standard for defining heatwaves globally, as definitions vary across communities, regions, and countries (Smith et al., 2013). According to the IMD, any day in India may be a candidate for a heatwave declaration when the maximum temperature of a station reaches at least 40 °C or more for plains and at least 30 °C or more for hilly regions. Heatwave decisions are made according to 2 criteria – based on a departure from the normal temperature, or an absolute temperature threshold. For departure from the normal, a heatwave is declared when the maximum temperature is more than 4.5 °C over the 30-year average historical maximum temperature. If the actual maximum temperature is above 45 °C, a heatwave is declared irrespective of the normal historical maximum temperature (IMD, 2023). Unfortunately, due to the unavailability of data on maximum temperature for all cities, we were unable to apply this definition to our results. However, when approximating the IMD definition by defining a heatwave based on 1-day, our study revealed that this definition might underestimate the association on mortality as lower estimates were found compared to the other heatwave definitions. This finding is consistent with literature suggesting that the severity of heatwaves is lower during the first day of exposure (Nori-Sarma et al., 2019a). Nonetheless, we still observed an increased risk of mortality when using a 1-day heatwave definition as a proxy for IMD guidelines, highlighting the significance of even a single extreme heat day as a public health concern with relevant policy implications. Identifying and monitoring single days of high temperature can be important for implementing locally relevant policies by developing effective heatwave plans and interventions to alleviate the burden of heatwaves on population health.

We additionally looked at potential effect modification of heatwave impacts by intensity, duration, and timing since the beginning of the summer season, and found modest evidence of effect modification. Interestingly, timing of occurrence of a heatwave was not an important effect modifier affecting mortality. By comparison, increasing intensity (measured as higher mean temperature values of the heatwave) appeared to have the strongest association with increasing risk of heatwave-related mortality when aggregated across all communities included within our study. The results were in line with a previous published study in Northwest India (Nori-Sarma et al., 2019a). This has important implications for policy, especially as average temperature trends continue to increase with continued climate change.

While heatwaves may be more damaging from a relative risk perspective, we additionally wanted to estimate the magnitude of the impact of different types of heatwaves from an absolute mortality perspective. Our results show that some heatwaves are rare but also carry increased risks of mortality; however, due to the rarity, the absolute impact in attributable mortality fraction is relatively low. We were able to calculate population-weighted attributable fractions by heatwave definition, showing the policy-relevant tradeoffs between number of days declared as heatwave periods versus the overall numbers of mortalities occurring as a result of those heatwaves. While we acknowledge that heatwaves represent only a portion of the broader impact of extreme temperatures, we chose to prioritize heatwaves due to their significant implications for public health. This focus has direct implications for policymakers and lays a foundation for future scientific work, including the development of effective heat-health warning system and who may be determining how to allocate resources during periods of extreme heat in India.

Our study faced some limitations. Firstly, we were unable to obtain maximum temperature data for all our study period, hindering us to replicate the heatwave definition provided by IMD. Second, there exists heterogeneity in the completeness of death registration across different states and cities in India, which may result in missed deaths within the civil registration system. We believe the missingness of these deaths is likely random in relation to heatwave definitions or occurrences and unlikely to introduce differential bias in our effect estimates; in fact, given the general trends in reporting of mortalities to relevant officials, we anticipate that our findings would be an underestimate of the risk of mortality due to heat (Nori-Sarma et al., 2019b). Thirdly, we further did not focus on interactions of air pollution in the associations between heatwaves and daily mortality as such analysis was beyond the scope of this paper. Given that India experiences extreme levels of both exposures, further investigation in this context is warranted. Finally, information was not available on crucial sociodemographic factors such as age, gender, ethnicity, and socioeconomic status, as well as other potential individual-level effect modifiers. As more detailed health data and contextual information becomes available, we hope that future studies will be able to explore how the impacts of heat vary across strata defined by individual (e.g., age, sex, occupation, access to air conditioning) or contextual (e.g., neighborhood socioeconomic markers) variables. These studies should identify possible sociodemographic disparities as heat-related inequalities are expected to be higher in more deprived communities as they are overcrowded, have less access to basic amenities, limited availability to health care, poorer housing conditions and less access to air conditioning (Dimitrova et al., 2021). Further, deprived individuals are more likely work outdoors exposed to extremer temperatures.

Despite these limitations, our study has many implications for policy, especially under a changing climate. At the recent COP28 convening, which included for the first time a “health ministry”, the importance of the health impacts of climate change were highlighted(WHO, 2023). Sharply increasing temperatures, including a 2023 heat wave season in India that started earlier than the norm and lasted longer across the country(CNN, 2023), underscores the importance of this work to assess impacts of heat waves on human health. Our findings have a variety of implications for policymakers sub-nationally, nationally and globally, and suggest opportunities for future research in the region. As the climate continues to change, areas that are currently in mid-latitudes with moderate temperatures may begin to experience more extreme temperatures (Hansen et al., 2006; Karl and Trenberth, 2003). Lessons learned in assessment of heatwave impacts on health in areas such as India, where extreme temperatures are already commonplace and health effects are dramatic, can guide development of heatwave alerts for areas that may begin to experience temperature extremes as the globe undergoes an overall warming with more temperature extremes. There are also opportunities for policy makers in India to continue to implement heatwave early warning systems including extreme temperature alerts and corresponding interventions to mitigate the health effects of extreme heat, especially given our findings that heatwave intensity is one of the primary effect modifiers of extreme heat health impacts. Heterogeneous results across cities indicate the value of multicity research and suggest that approaches to prevent weather-related mortality might be most effective if they are city specific.

## 5. Conclusion

In this large time-series study in India we observed strong effects of heatwaves on daily mortality. We observed that longer and more intense heatwaves are linked to an increased mortality risk, whereas using shorter and less intense definitions of heatwaves resulted in a higher burden of heatwave-related deaths. Both definitions of heatwaves and the burden associated with each definition should be incorporated into planning and decision-making processes for policymakers to effectively prioritize public health interventions that address the present and future health risks associated with heatwaves in India.

## Supplementary Material

Supplement file

## Figures and Tables

**Table 1:** Heatwave characteristics by city under the definition of 2 consecutive days above the 97th percentile.

City	Time period	Daily deaths (mean)	N° of heatwaves	Heatwaves per year (mean)	Day in the season from start summer^¶^ (mean days)	Length heatwave (mean days)	Intensity heatwave (mean heatwave temperature in °C and (%)) ^#^
Ahmedabad	2008 - June 2019	122	28	3.1	83.1	4.1	36.8 (2.4)]
Bangalore	2008—2012	121	13	3.2	45.2	3.8	29.8 (1.9)]
Chennai	2010—2019	164	18	2.2	91.7	5.4	34.2 (1.5)]
Delhi	2011—2018	284	19	2.7	66.9	3.9	37.7 (3.0)]
Hyderabad	2008 - June 2011	78	8	4.0	69.6	4.9	35.2 (1.4)]
Kolkata	2010—2019	172	21	3.0	74.0	4.2	34.2 (1.8)]
Mumbai	2009 – Nov. 2015	251	19	3.6	98.7	3.7	31.5 (1.1)]
Pune	2008—2012	68	13	4.3	51.2	3.8	31.3 (2.4)]
Shimla	2008 – Aug. 2012	5	10	2.5	100.7	4.8	26.5 (3.8)]
Varanasi	2008 – Nov 2018*	23	20	2.2	61.5	4.8	37.6 (2.1)]
**Overall**	**2008–2019**	**136**	**168**	**2.9**	75.2	**4.3**	**34.2 (2.1)**

* No data available in 2017 for Varanasi.

# Intensity (%): estimated as the percentage increase of the average daily mean temperature of the specific heatwave compared to the 97th percentile of the specific city.

¶ The start of the summer season was considered 1st of March for all cities except Delhi and Varanasi which was 1st of April.

**Table 2:** Effect modification by each of the potential modifiers – length, intensity, day in summer season.

City	Length (Change per 1 day increase in duration)	Intensity (Change per 1 % increase in temperature)	Day in summer season (Change per 1 day later start of heatwave)
**Individual model**	1.4 % (0.7 %; 2.0 %)	3.5 % (2.7 %; 4.3 %)	0.0 % (−0.0 %; 0.0 %)
**Mutually adjusted model** ^#^	− 0.2 % (−1.0 %; 0.5 %)	3.8 % (2.8 %; 4.7 %) ^*^	0.1 % (−0.0 %; 0.1 %)

* Interpretation: one % unit increase of mean temperature above the 2-day 97th centile was associated with a 3.8% increase (95%CI, 2.8; 4.7) in the estimated association between heatwaves and mortality.

# We evaluated each characteristic individually in single exposure models and mutually adjusted all characteristics in one multiple exposure model in order to evaluate the independent effect of each characteristic.

## Data Availability

The data that has been used is confidential.
