# Supplementary material for: Impact of heatwaves on all-cause mortality in India: A comprehensive multi-city study
Source: Environ Int. Author manuscript; Available in PMC 2025 Feb 3. (PMC11790314; doi:10.1016/j.envint.2024.108461)
Supplement: Supplement file [file NIHMS2045245-supplement-Supplement_file.docx]

**Impact of heatwaves on mortality in India: a comprehensive multi-city study**

Jeroen de Bont^1,^*, Amruta Nori-Sarma^2,^*, Massimo Stafoggia^1,3^, Tirthankar Banerjee^4^, Vijendra Ingole^5^, Suganthi Jaganathan^1,6,7^, Siddhartha Mandal^6,7^, Ajit Rajiva^6,7^, Bhargav Krishna^8^, Itai Kloog^9,10^, Kevin Lane^2^, Rajesh K Mall^11^, Abhiyant Suresh Tiwari^12^, Yaguang Wei^13^, Gregory A. Wellenius^2^, Dorairaj Prabhakaran^6,7^, Joel Schwartz^13,#^, Poornima Prabhakaran^6,7,#^, Petter Ljungman^1,14,#^.

^1^ Institute of Environmental Medicine, Karolinska Institutet, Stockholm, Sweden

^2^ Center for Climate and Health, Boston University School of Public Health, Boston, MA

^3^ Department of Epidemiology, Lazio Region Health Service /ASL Roma 1, Rome, Italy

^4^ Institute of Environment and Sustainable Development, Banaras Hindu University, Varanasi, India

^5^ Office for National Statistics, Wales, Newport, United Kingdom

^6^ Centre for Chronic Disease Control, New Delhi, India

^7^ Ashoka University, Sonipat, India

^8^ Centre for Policy Research, New Delhi, India

^9^ Ben-Gurion University of the Negev, Beer-Sheva, Israel

^10^ Department of Environmental Medicine and Public Health, Icahn School of Medicine at Mount Sinai, New York, NY, USA

^11^ DST-Mahamana Center of Excellence in Climate Change Research, Institute of Environment and Sustainable Development Banaras Hindu University, Varanasi, India

^12^NRDC India, New Delhi, India

^13^ Department of Environmental Health, Harvard T.H. Chan School of Public Health, Boston, MA, USA

^14^ Department of Cardiology, Danderyd Hospital, Stockholm, Sweden

* Shared first authorship

^#^ shared senior authorship

**Table S1:** Percent increase (95% CI) in mortality risk due to multiple heatwaves definitions (same results as in Fig. 2 of the manuscript).

**Table S2:** Effect modification by each of the potential modifiers – length, intensity, day in summer season.

**Table S3:** Comparison of attributable deaths comparing the heatwave duration and intensity by city. (same results as in Fig. 3 of the manuscript)

**Figure S1:** City-specific heatwave duration, intensity, timing in season for each heatwave.

**Figure S2:** Sensitivity analyses: comparing mean vs maximum temperature using 58% of the dataset.

**Figure S3:** Sensitivity analyses: different adjustments evaluating the association between heatwaves and daily mortality.

**Table S1:** Percent increase (95% CI) in mortality risk due to multiple heatwaves definitions (same results as in Fig. 2 of the manuscript).

| 1. **Percentile 95^th^** | | | | |  |
| --- | --- | --- | --- | --- | --- |
| **City** | **1 cons. day** | **2 cons. day** | **3 cons. day** | **5 cons. day** | |
| Ahmedabad | 18.4 (15.7;21.1) | 22.1 (19.3;24.9) | 24.9 (22.0;27.9) | 26.9 (23.5;30.5) | |
| Bangalore | 5.4 (2.6;8.4) | 4.8 (1.6;8.1) | 4.4 (0.8;8.1) | 3.5 (-0.8;8.1) | |
| Chennai | 13.9 (12.0;15.8) | 16.8 (14.6;19.1) | 18.8 (16.2;21.4) | 21.0 (17.7;24.5) | |
| Delhi | 9.7 (7.7;11.8) | 12.5 (10.1;14.9) | 15.6 (12.8;18.4) | 17.1 (13.7;20.6) | |
| Hyderabad | 7.8 (3.1;12.7) | 13.4 (8.0;19.1) | 15.9 (10.0;22.2) | 10.2 (3.2;17.7) | |
| Kolkata | 11.1 (9.2;13.1) | 12.4 (10.2;14.7) | 14.1 (11.6;16.7) | 11.8 (8.9;14.9) | |
| Mumbai | 5.1 (3.2;7.0) | 3.8 (1.6;5.9) | 3.2 (0.8;5.6) | 1.4 (-1.4;4.3) | |
| Pune | 8.6 (4.6;12.7) | 10.3 (5.9;14.9) | 11.6 (6.6;16.8) | 15.3 (9.0;22.1) | |
| Shimla | -0.7 (-13.1;13.4) | 9.7 (-5.8;27.7) | 13.3 (-4.4;34.3) | 34.1 (9.3;64.7) | |
| Varanasi | 19.5 (14.2;25.0) | 21.4 (15.5;27.6) | 24.9 (18.3;31.8) | 29.2 (21.5;37.5) | |
| **Pooled Estimate, I^2^** | **10.5 (7.4;13.6), 92%** | **12.7 (8.9;16.6), 94%** | **14.4 (9.9;19.2), 95%** | **15.5 (9.7;21.5), 95%** | |

| 1. **Percentile 97^th^** | | | | |  |
| --- | --- | --- | --- | --- | --- |
| **City** | **1 cons. day** | **2 cons. day** | **3 cons. day** | **5 cons. day** | |
| Ahmedabad | 20.9 (18.0;23.7) | 24.9 (21.7;28.2) | 31.0 (27.2;34.9) | 42.4 (37.1;47.8) | |
| Bangalore | 2.9 (-0.5;6.4) | 5.7 (1.6;9.9) | 4.7 (-0.1;9.7) | 1.9 (-4.6;8.8) | |
| Chennai | 17.5 (15.1;19.9) | 20.5 (17.7;23.3) | 22.0 (18.8;25.2) | 24.4 (20.4;28.4) | |
| Delhi | 12.5 (10.0;15.0) | 16.6 (13.5;19.7) | 20.5 (16.8;24.4) | 24.0 (18.7;29.7) | |
| Hyderabad | 13.9 (8.1;20.1) | 16.4 (10.0;23.2) | 17.6 (10.4;25.3) | 11.2 (2.7;20.5) | |
| Kolkata | 11.2 (8.9;13.6) | 15.3 (12.4;18.3) | 17.8 (14.3;21.3) | 15.4 (10.6;20.4) | |
| Mumbai | 5.2 (3.0;7.4) | 3.4 (0.8;6.1) | 4.0 (0.9;7.2) | 5.0 (0.3;10.0) | |
| Pune | 10.2 (5.4;15.2) | 11.0 (5.6;16.8) | 14.3 (7.9;21.1) | 12.1 (2.1;23.1) | |
| Shimla | 6.0 (-9.6;24.2) | 8.3 (-9.3;29.2) | 17.5 (-3.5;43.0) | 32.0 (4.2;67.1) | |
| Varanasi | 19.2 (13.0;25.6) | 23.1 (16.0;30.7) | 32.2 (23.8;41.2) | 38.0 (27.4;49.5) | |
| **Pooled Estimate, I^2^** | **12.2 (8.5;15.9), 93%** | **14.7 (10.3;19.3), 94%** | **17.8 (12.1;23.8), 94%** | **19.4 (11.5;27.9), 94%** | |

| 1. **Percentile 99^th^** | | | | |  |
| --- | --- | --- | --- | --- | --- |
| **City** | **1 cons. day** | **2 cons. day** | **3 cons. day** | **5 cons. day** | |
| Ahmedabad | 27.1 (23.0;31.4) | 46.9 (40.7;53.4) | 55.8 (47.1;65.0) | 41.8 (26.8;58.7) | |
| Bangalore | 3.9 (-1.2;9.2) | 4.6 (-2.2;11.7) | 8.7 (-0.6;18.8) | - | |
| Chennai | 17.8 (14.3;21.5) | 19.2 (14.9;23.6) | 25.6 (20.1;31.4) | 34.7 (25.5;44.7) | |
| Delhi | 17.5 (13.6;21.6) | 21.6 (16.5;26.9) | 23.8 (17.3;30.6) | 29.6 (11.0;51.5) | |
| Hyderabad | 13.0 (3.9;22.8) | 15.7 (5.3;27.1) | 22.7 (10.1;36.7) | 39.3 (10.3;76.0) | |
| Kolkata | 14.3 (10.6;18.1) | 17.9 (12.9;23.1) | 14.0 (8.0;20.3) | 4.2 (-3.7;12.8) | |
| Mumbai | 3.1 (-0.2;6.6) | 4.7 (-0.3;10.1) | 9.8 (1.0;19.3) | - | |
| Pune | 9.3 (2.3;16.7) | 5.4 (-4.1;15.9) | 2.7 (-11.9;19.7) | - | |
| Shimla | 19.9 (-4.5;50.7) | 42.8 (10.0;85.4) | 27.6 (-12.7;86.4) | - | |
| Varanasi | 27.8 (18.3;38.2) | 36.6 (24.0;50.4) | 44.0 (28.6;61.3) | 63.3 (42.6;87.0) | |
| **Pooled Estimate, I^2^** | **14.7 (9.5;20.1), 92%** | **19.5 (11.3;28.4), 94%** | **22.7 (13.3;32.8), 91%** | **33.3 (18.6;49.8), 89%** | |

Estimates are provided as percentage change in mortality and 95% confidence interval comparing heatwaves vs non-heatwave days. Models were adjusted for a penalized spline smooth function of calendar day with nine degrees of freedom (*df*), an indicator of day-of-week, a natural spline function with 4 *df* for adjusted dew point temperature (lag 0-1, and air pollution (lag 0-1). I^2^ is obtained from the heteorogeneity test indicating the percentage of variability in results across studies. Heatwaves above the 99^th^ percentile for 5 consecutive days was estimated including 6 cities (Ahmedabad, Chennai, Delhi, Hyderabad, Kolkata, Varanasi)

**Table S2.** Effect modification by each of the potential modifiers – length, intensity, day in summer season.

| **City** | **Length**  **(Change per 1 day increase in duration)** | |  | **Intensity**  **(Change per 1 % increase in temperature)** | |  | **Day in season**  **(Change per 1 day later start of heatwave)** | |
| --- | --- | --- | --- | --- | --- | --- | --- | --- |
|  | **Individual** | **Combined** |  | **Individual** | **Combined** |  | **Individual** | **Combined** |
| Ahmedabad | 2.19 (-0.79; 5.16) | 1.39 (-1.47; 4.24) |  | 3.40 (-0.39; 7.20) | 2.42 (-1.08; 5.91) |  | 0.55 (0.15; 0.96) | 0.50 (0.12; 0.88) |
| Bangalore | 0.31 (-4.22; 4.83) | 0.22 (-4.62; 5.07) |  | 1.13 (-4.00; 6.25) | 1.15 (-4.31; 6.60) |  | 0.19 (-0.19; 0.56) | 0.19 (-0.18; 0.57) |
| Chennai | 0.45 (-2.17; 3.07) | 0.47 (-2.45; 3.39) |  | 0.09 (-8.03; 8.21) | -0.71 (-9.76; 8.33) |  | -0.26 (-0.67; 0.15) | -0.26 (-0.67; 0.15) |
| Delhi | 3.07 (-1.49; 7.63) | 1.94 (-3.25; 7.14) |  | 2.81 (-1.22; 6.85) | 1.70 (-3.10; 6.50) |  | 0.21 (-0.25; 0.66) | 0.09 (-0.39; 0.58) |
| Hyderabad | 2.33 (-1.26; 5.93) | 3.58 (-3.30; 10.47) |  | 0.56 (-8.00; 9.13) | -5.71 (-20.88; 9.47) |  | -0.35 (-0.71; 0.01) | -0.20 (-0.67; 0.27) |
| Kolkata | 2.63 (-1.27; 6.53) | 2.82 (-1.13; 6.77) |  | -1.05 (-6.52; 4.42) | -1.70 (-7.31; 3.91) |  | -0.00 (-0.35; 0.34) | -0.01 (-0.36; 0.34) |
| Mumbai | 0.37 (-4.98; 5.71) | 0.20 (-5.19; 5.59) |  | 3.74 (-12.28; 19.77) | 4.98 (-12.60; 22.55) |  | 0.01 (-0.13; 0.15) | 0.03 (-0.12; 0.18) |
| Pune | 1.57 (-3.37; 6.51) | -0.03 (-4.44; 4.38) |  | 6.80 (2.19; 11.41) | 6.83 (1.95; 11.70) |  | -0.01 (-0.60; 0.58) | -0.08 (-0.55; 0.40) |
| Shimla | 0.79 (-1.98; 3.56) | -2.14 (-5.96; 1.69) |  | 2.80 (-0.37; 5.97) | 4.79 (-0.23; 9.81) |  | -0.28 (-0.81; 0.25) | -0.10 (-0.58; 0.38) |
| Varanasi | 3.15 (0.81; 5.50) | 2.34 (-0.31; 4.99) |  | 8.36 (1.75; 14.96) | 1.91 (-5.71; 9.53) |  | 0.67 (0.16; 1.18) | 0.53 (0.05; 1.02) |
| **All** | **1.36 (0.71; 2.01)** | **-0.24 (-1.00; 0.53)** |  | **3.51 (2.72; 4.31)** | **3.75 (2.80; 4.69)** |  | **0.03 (-0.03; 0.09)** | **0.06 (-0.00; 0.12)** |

**Table S3:** Comparison of attributable deaths comparing the heatwave duration and intensity by city. (same results as in Fig. 3 of the manuscript).

| 1. **Percentile 95^th^** | | | | |
| --- | --- | --- | --- | --- |
| **City** | **1 cons. day** | **2 cons. day** | **3 cons. day** | **5 cons. day** |
| Ahmedabad | 328 (287;368) | 315 (281;347) | 292 (264;320) | 217 (195;240) |
| Bangalore | 114 (55;172) | 73 (25;119) | 49 (9;88) | 25 (-6;54) |
| Chennai | 362 (317;405) | 307 (272;341) | 244 (215;272) | 164 (142;185) |
| Delhi | 463 (372;553) | 422 (350;493) | 374 (316;430) | 244 (202;286) |
| Hyderabad | 107 (44;167) | 139 (87;188) | 129 (85;170) | 51 (17;82) |
| Kolkata | 312 (261;361) | 260 (217;302) | 233 (196;270) | 138 (106;169) |
| Mumbai | 187 (120;254) | 95 (43;147) | 61 (16;104) | 16 (-16;47) |
| Pune | 71 (40;101) | 66 (39;92) | 60 (36;83) | 43 (26;58) |
| Shimla | -0 (-9;7) | 3 (-2;9) | 3 (-1;6) | 3 (1;4) |
| Varanasi | 65 (50;80) | 53 (40;65) | 47 (36;57) | 34 (27;42) |
| **Pooled Estimate** | **2009 (1538;2468)** | **1732 (1352;2102)** | **1492 (1172;1801)** | **935 (693;1167)** |

| 1. **Percentile 97^th^** | | | | |
| --- | --- | --- | --- | --- |
| **City** | **1 cons. day** | **2 cons. day** | **3 cons. day** | **5 cons. day** |
| Ahmedabad | 219 (193;243) | 178 (159;196) | 146 (132;159) | 102 (92;110) |
| Bangalore | 38 (-6;80) | 47 (14;78) | 25 (-1;49) | 5 (-13;21) |
| Chennai | 266 (235;297) | 221 (196;245) | 181 (160;203) | 134 (116;151) |
| Delhi | 348 (285;409) | 278 (233;322) | 218 (184;251) | 110 (90;130) |
| Hyderabad | 124 (76;170) | 114 (74;152) | 90 (57;121) | 34 (9;57) |
| Kolkata | 192 (156;229) | 163 (135;190) | 129 (107;150) | 55 (39;69) |
| Mumbai | 115 (67;162) | 48 (12;84) | 35 (8;61) | 19 (1;36) |
| Pune | 53 (30;76) | 32 (17;46) | 24 (14;34) | 7 (1;12) |
| Shimla | 2 (-4;7) | 2 (-2;5) | 2 (-1;5) | 2 (0;3) |
| Varanasi | 41 (29;51) | 34 (25;42) | 33 (26;39) | 23 (18;27) |
| **Pooled Estimate** | **1397 (1061;1723)** | **1116 (861;1361)** | **883 (686;1073)** | **490 (354;618)** |

| 1. **Percentile 99^th^** | | | | |
| --- | --- | --- | --- | --- |
| **City** | **1 cons. day** | **2 cons. day** | **3 cons. day** | **5 cons. day** |
| Ahmedabad | 94 (82;105) | 70 (64;77) | 43 (39;48) | 10 (7;12) |
| Bangalore | 17 (-6;39) | 11 (-5;25) | 10 (-1;19) | - |
| Chennai | 91 (75;106) | 63 (51;74) | 46 (38;54) | 21 (16;25) |
| Delhi | 159 (128;190) | 107 (86;128) | 68 (53;83) | 8 (4;12) |
| Hyderabad | 39 (13;63) | 32 (12;50) | 29 (14;42) | 7 (2;11) |
| Kolkata | 87 (67;107) | 59 (44;73) | 31 (19;43) | 5 (-5;13) |
| Mumbai | 24 (-2;49) | 14 (-1;28) | 8 (1;15) | - |
| Pune | 16 (4;28) | 7 (-6;18) | 2 (-9;11) | - |
| Shimla | 1 (-0;3) | 1 (0;2) | 0 (-0;1) | - |
| Varanasi | 18 (13;23) | 14 (10;18) | 11 (8;13) | 7 (5;8) |
| **Pooled Estimate** | **547 (375;712)** | **378 (255;493)** | **248 (162;328)** | **58 (30;82)** |

**Figure S1:** City-specific heatwave duration, intensity, timing in season for each heatwave.

| 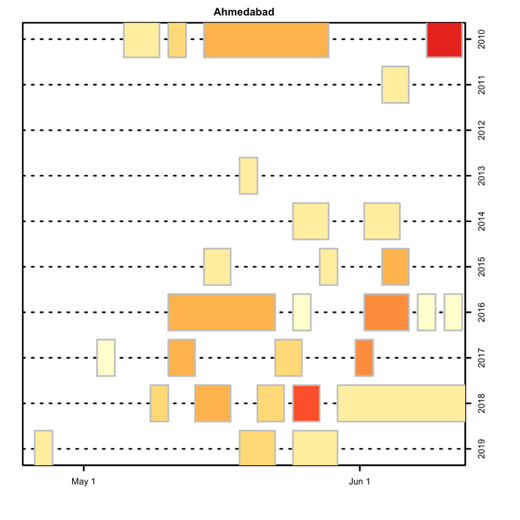 | 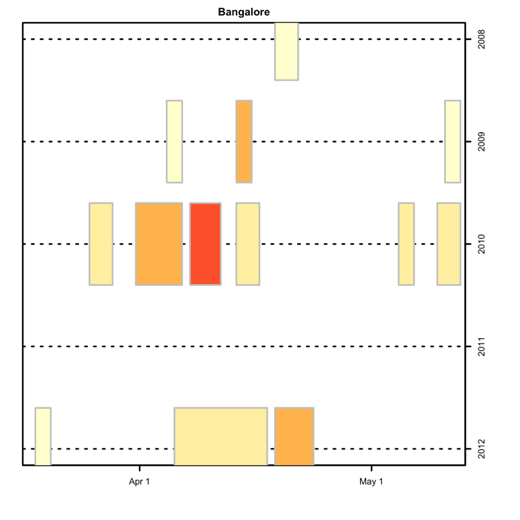 | 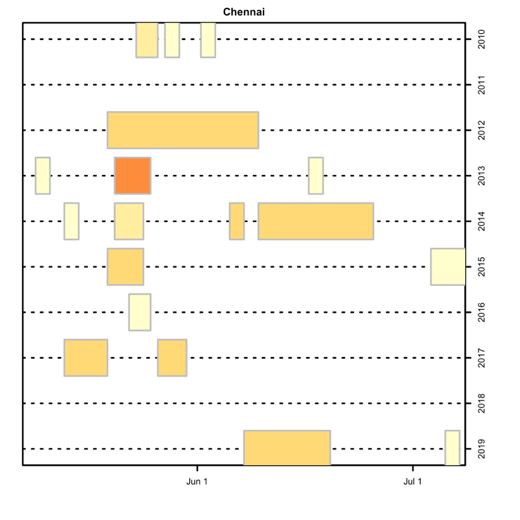 | 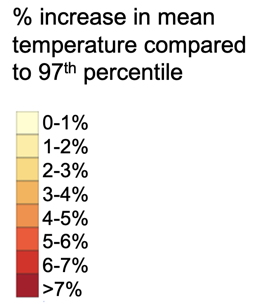 |
| --- | --- | --- | --- |
| 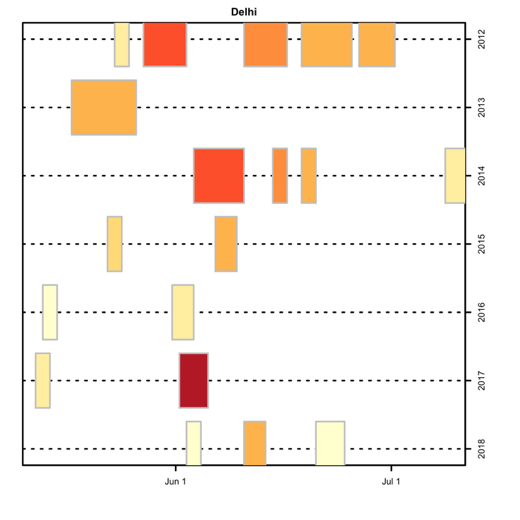 | 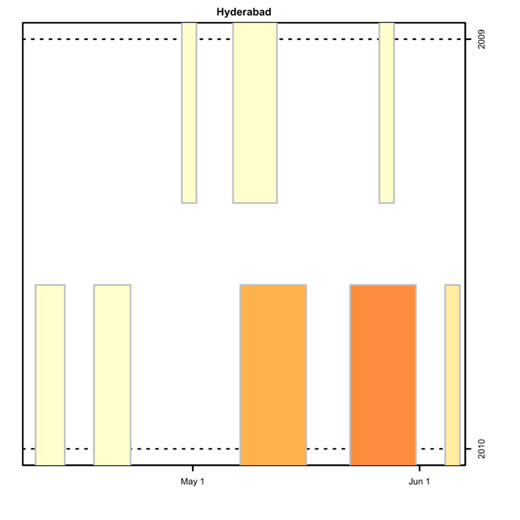 | 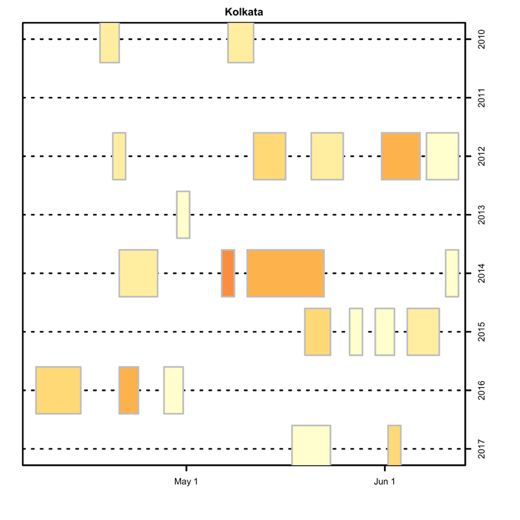 |  |
| 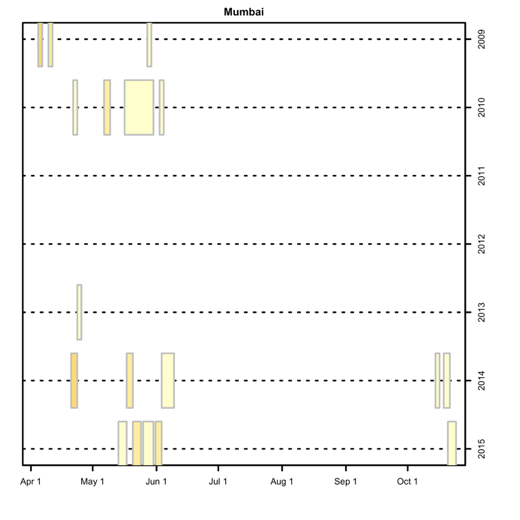 | 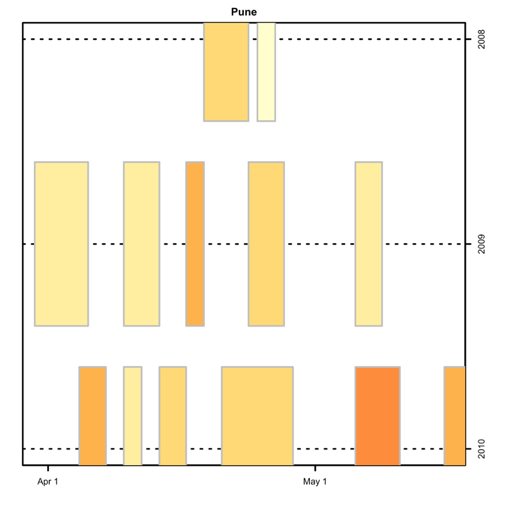 | 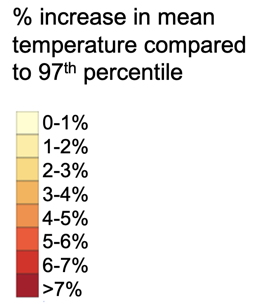 |  |
| 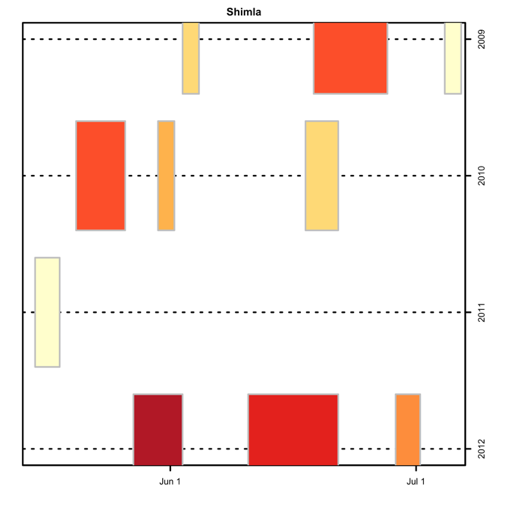 | 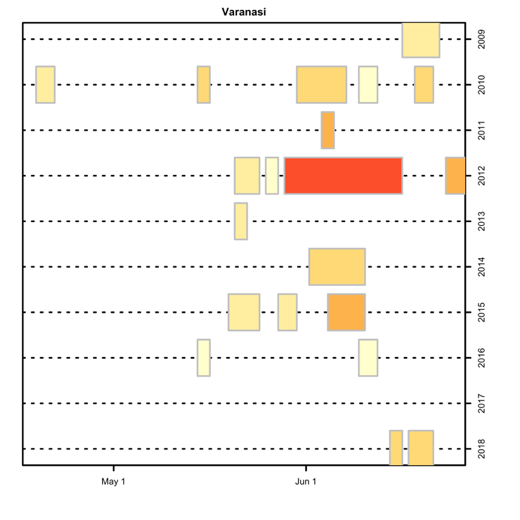 |  |  |

**Figure S2:** Sensitivity analyses: comparing mean vs maximum temperature using 58% of the dataset.


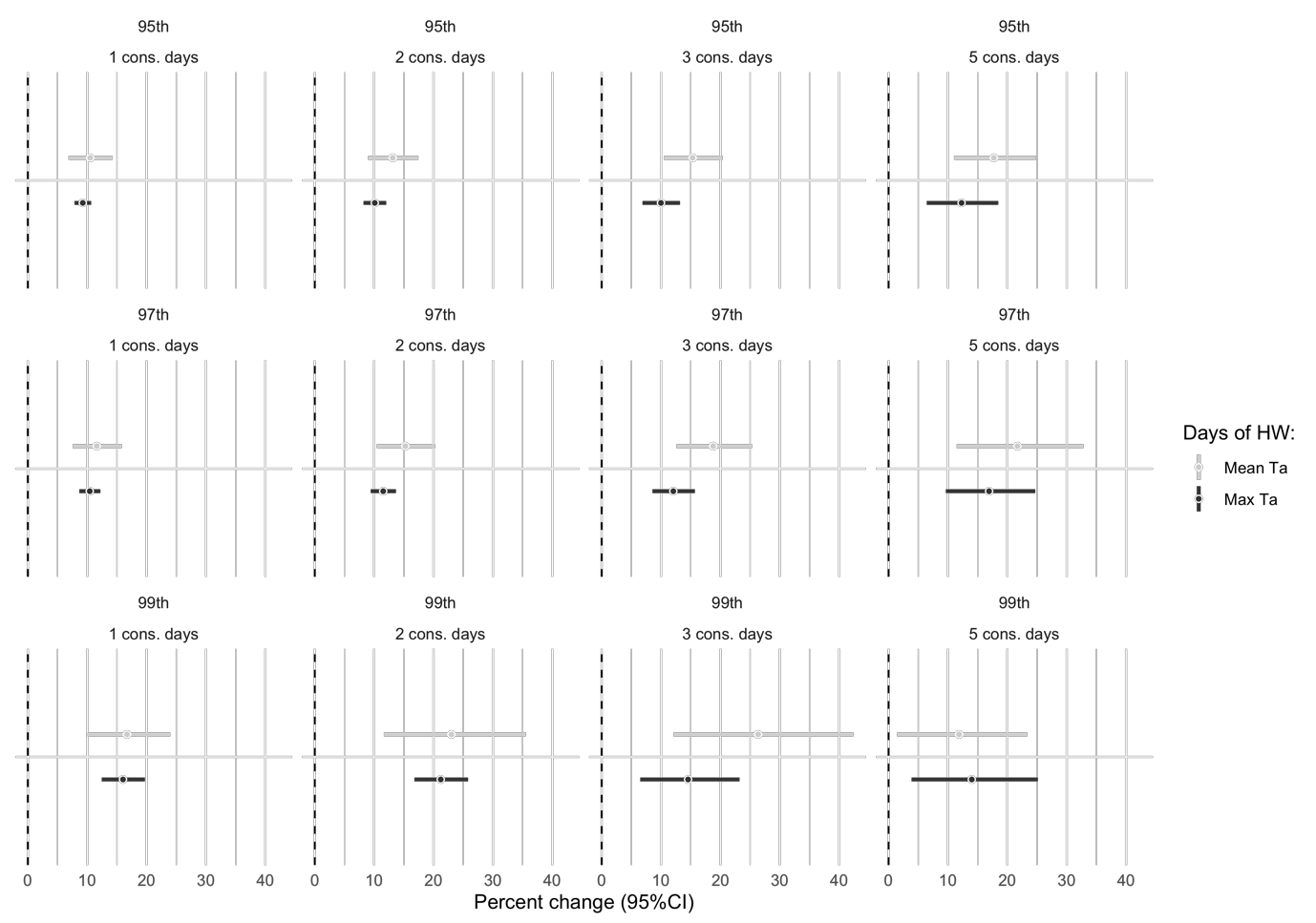


Note: estimates are provided as percentage change in mortality and 95% confidence interval comparing heatwaves vs non-heatwave days. Models were adjusted for a penalized spline smooth function of calendar day with nine degrees of freedom (*df*), an indicator of day-of-week, a natural spline function with 4 *df* for adjusted dew point temperature (lag 0-1, and air pollution (lag 0-1).

**Figure S3:** Sensitivity analyses: different adjustments evaluating the association between heatwaves and daily mortality.


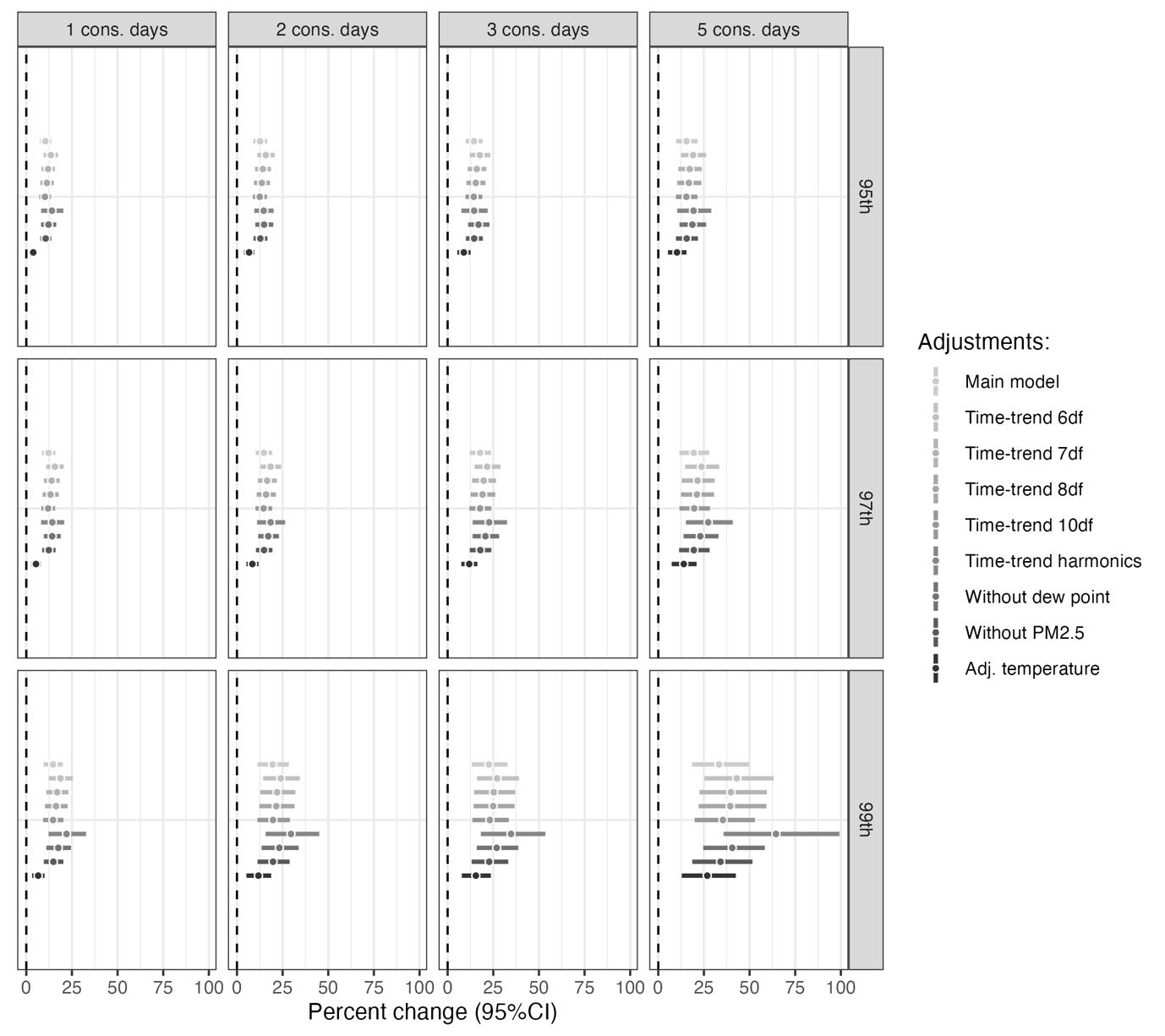


Estimates are given as percentage change in mortality and 95% confidence interval comparing heatwave vs non heatwave days. The main model evaluated time trends with 9 df. We evaluated different degrees of freedoms adjusting for time trends (6-10 df/year). We additionally evaluated the effects without adjusted dew point temperature or PM_2.5_. In the last model we additionally adjusted for air temperature (lag0).
